# Clinical indications and patient outcomes of intracranial venous sinus stenting beyond overt idiopathic intracranial hypertension: a scoping review

**DOI:** 10.1007/s00701-025-06514-7

**Published:** 2025-04-25

**Authors:** Shiva A. Nischal, Rocío Fernández-Méndez, Vasu Gautam, Shaan Patel, Catherine J. McMahon, Peter J. Hutchinson, John D. Pickard, J. Nicholas P. Higgins, Alexis J. Joannides

**Affiliations:** 1 School of Clinical Medicine, University of Cambridge, Cambridge, UK; 2 Department of Clinical Neurosciences, Addenbrooke’s Hospital, Cambridge, UK; 3 Present Address: Department of Physiology, Anatomy & Genetics, Medical Sciences Division, University of Oxford, Oxford, UK; 4 Faculty of Health Sciences, Universitat Jaume I, Castelló, Spain; 5 Department of Neurosurgery, The Walton Centre, Liverpool, UK; 6 NIHR HealthTech Research Centre for Brain Injury, Cambridge, UK; 7 Department of Radiology, Addenbrooke’s Hospital, Cambridge, UK

**Keywords:** Venous sinus stenting, Neurosurgery, Endovascular

## Abstract

**Background:**

Intracranial venous sinus stenting (VSS) was initially developed as an alternative approach to addressing venous outflow obstruction in the context of idiopathic intracranial hypertension (IIH). In recent years, the technique has been increasingly used for other conditions involving venous compromise beyond overt IIH. The aim of this study was to describe the nature and volume of literature considering clinical applications and efficacy of VSS.

**Methods:**

A scoping review was conducted using MEDLINE, EMBASE, Scopus, The Cochrane Library, and various grey literature sources. Articles published since the introduction of VSS in 2002 were included. Independent screening of articles occurred in two stages: title-and-abstract and full-text screening. Relevant data was extracted and evidence mapping with narrative synthesis followed.

**Results:**

The search strategy yielded 1814 articles, of which 165 were included in this review. A total of 27 additional clinical indications of VSS beyond overt IIH were identified, spanning a diverse range of neurological pathology. Most evidence came from case reports, with the United States being the commonest study origin. Focal stenotic lesions and stenting locations were distributed throughout the dural sinus anatomy. An outline of patient outcomes reported by VSS providers is presented, with pulsatile tinnitus and visual impairment showing the greatest likelihood of clinical resolution.

**Conclusion:**

This scoping review demonstrates the wider clinical utility and therapeutic potential of VSS beyond overt IIH. We also highlight the need for further studies to assess efficacy for each respective indication and clinical standardisation of VSS practice.

**Supplementary Information:**

The online version contains supplementary material available at 10.1007/s00701-025-06514-7.

## Introduction

Intracranial venous sinus stenting (VSS) is a minimally invasive endovascular procedure which was initially developed as a therapeutic option for idiopathic intracranial hypertension (IIH) [15]. Insertion of a metallic stent in focal stenotic lesions of the transverse sinus helped resolve venous hypertension, thought to be a key determinant in the pathophysiology of IIH [18]. By definition, IIH has no known cause and is the primary form of the pseudotumor cerebri syndrome (PTCS) characterised as a syndrome of increased cerebrospinal fluid (CSF) pressure without ventriculomegaly, mass lesion, or meningeal abnormality [11, 22]. IIH most commonly affects overweight women of childbearing age, with patients typically complaining of new-onset headaches, brief episodes of unilateral or bilateral visual disturbance (including loss of vision, double vision, and decreased visual acuity), venous pulsatile tinnitus (VPT), fatigue, and low mood. Papilloedema and raised CSF pressures are usually also present. The evidence base in support of VSS as a safe, less invasive, and efficacious alternative to surgical approaches, such as optic nerve sheath fenestration or CSF shunting, in the treatment of IIH is growing considerably [14, 25].

However, the application of VSS to clinical indications beyond full-blown IIH is not well characterised, and the relevance of cerebral venous congestion in these broader indications is now widely debated [2, 35]. In principle, VSS can be used to treat intracranial venous hypertension secondary to venous outflow obstruction and venous sinus stenosis. A number of conditions are known to involve compromised dural venous outflow, including venous sinus thrombosis and invasion by meningioma, but the clinical efficacy of VSS in these diverse indications is unclear [16, 20]. Considerable variability also exists within IIH, with the possibility for diagnostic heterogeneity becoming increasingly recognised [10]. For instance, CSF pressure may not always be elevated in IIH, nor is papilloedema always present, and in cases where IIH itself leads to a CSF leak and subsequent spontaneous intracranial hypotension, increased CSF pressure may only become apparent once the leak is sealed. However, rebound headache may develop after sealing the leak which is worse in patients restriction of cerebral venous outflow rather than raised CSF pressure per se [32]. Another example is VPT, which is commonly associated with IIH, but can also result from sigmoid sinus wall anomalies (SSWAs) (including sigmoid sinus diverticula and sigmoid plate dehiscence or thinning), stenosis of the transverse or sigmoid sinus (caused for example by chronic sinus thrombosis or enlarged arachnoid granulations), turbulent flow through emissary veins, and jugular vein abnormalities. Notably, in the majority of such cases, neither BMI nor CSF pressure exceeds the threshold for IIH (> 25 cmH_2_O) [12, 23]. While further research is required to confirm the true extent of this diagnostic heterogeneity, it is clear that VSS is clinically indicated in conditions beyond IIH and is already being used in cases with identifiable aetiology. In this review, the term ‘overt IIH’ has been used to exclude patients with the full-blown condition. Of course, there will probably be cases where subclinical IIH might have been present in addition to the primary venous lesions stented. However, the target of stenting in patients covered by this review was primarily venous obstruction, not milder forms of underlying IIH.

Venous sinus stenosis is initially diagnosed via non-invasive imaging such as magnetic resonance venography (MRV) or computed tomographic venography (CTV), but criteria for VSS intervention often require catheter venography/manometry to demonstrate a trans-stenotic pressure gradient (TSPG). The procedure typically involves a stent being advanced endovascularly across the stenotic lesion under general anaesthesia, with a period of antiplatelet therapy often used post-procedure to minimise the risk of in-stent thrombosis [4]. Modifications to the core procedural methodology (e.g. route of venous access, stent material, choice of antiplatelet) may be necessary depending on the patient’s anatomical configuration and indication for VSS, though broad consensus is yet to be reached.

The aims of this review were to identify the nature and volume of literature considering the use of VSS in clinical indications beyond overt IIH and generate a descriptive overview of the evidence on this subject.

## Methods

This scoping review was conducted in accordance with the JBI scoping review guide (2020) and reported following the Preferred Reporting Items for Systematic Reviews and Meta-Analyses—Scoping Review extension (PRISMA-ScR) [1, 33] (Supplementary Table 1). The review was guided by the questions: What are the clinical indications of VSS beyond IIH? What are the associated patient outcomes post-VSS? What is the nature and volume of supporting literature? A scoping review was the most appropriate methodology for our research question due to the anticipated heterogeneity of included studies and the breadth of the research topic. A protocol was published *a priori* on Open Science Framework.

### Search and screening process

Our search was conducted in MEDLINE (Ovid), EMBASE (Ovid), Scopus, The Cochrane Library and various grey literature sources (including Open Grey, Prevention Information and Evidence Library, The Grey Literature Report, The National Institute for Health and Care Excellence, International Clinical Trials Registry Platform (ICTRP), ClinicalTrials.gov and ISRCTN). Reference and citation lists of all included articles were manually examined to identify additional articles that met the inclusion criteria.

Search strategies utilised Medical Subject Headings (MeSH) terms in MEDLINE (or analogous operators in other databases), truncation markers, proximity searching, and Boolean operators. An example search strategy for MEDLINE (Ovid) is provided in Supplementary Table 2. Searches in the remaining datasets were adapted as appropriate. The full search was continuously updated until immediately prior to final analysis (15th August 2022). Before the analyses, duplicates were identified and removed using EndNote 20 (*Clarivate Analytics, PA, USA*).

Screening of articles was carried out by two independent reviewers according to pre-specified selection criteria in two stages: first, titles and abstracts were screened for relevance, and subsequently selected publications were further screened for eligibility through full-text review. Inclusion criteria were articles published in English and from 2002 onwards (which is when VSS was first reported in the literature) [15]. Exclusion criteria were articles not considering the following: the population (humans), concept (VSS) or context (clinical indication beyond overt IIH) of interest. No exclusion criteria were applied on the basis of study design. Any disagreements that arose between reviewers were resolved through discussion or consulting a third reviewer. Articles meeting the inclusion criteria of this review were also identified through manual examination of references and citations from screened articles.

### Data extraction

Two independent reviewers extracted the following data from the included studies and entered them into a customised data extraction form developed by the reviewers *a priori*: article identifiers (study title; first author; year of publication; country); study characteristics (where appropriate) (design; aims/purpose; population); results (clinical indication(s) for VSS; location and number of stenoses; location and number of stents placed; measurement of pre-stenting TSPG, proximal venous sinus pressure, and intracranial CSF pressure; outcomes (including complications). Where necessary, corresponding authors were contacted directly to obtain further unpublished information, missing data or to clarify published information that could be pertinent to the review.

### Data analysis

Results from data extraction were collated, summarised, and presented via evidence mapping (tabulation and figure generation) accompanied by a narrative overview to describe the data and explain how the results relate to the review questions. GraphPad Prism 10.0.3 (*GraphPad Software, MA, USA*) was used for graphing, and Tableau 2023.2 (*Tableau Software, WA, USA*) was used to generate a heat map.

## Results

### Global epidemiology of venous sinus stenting practice

**Fig. 1:**
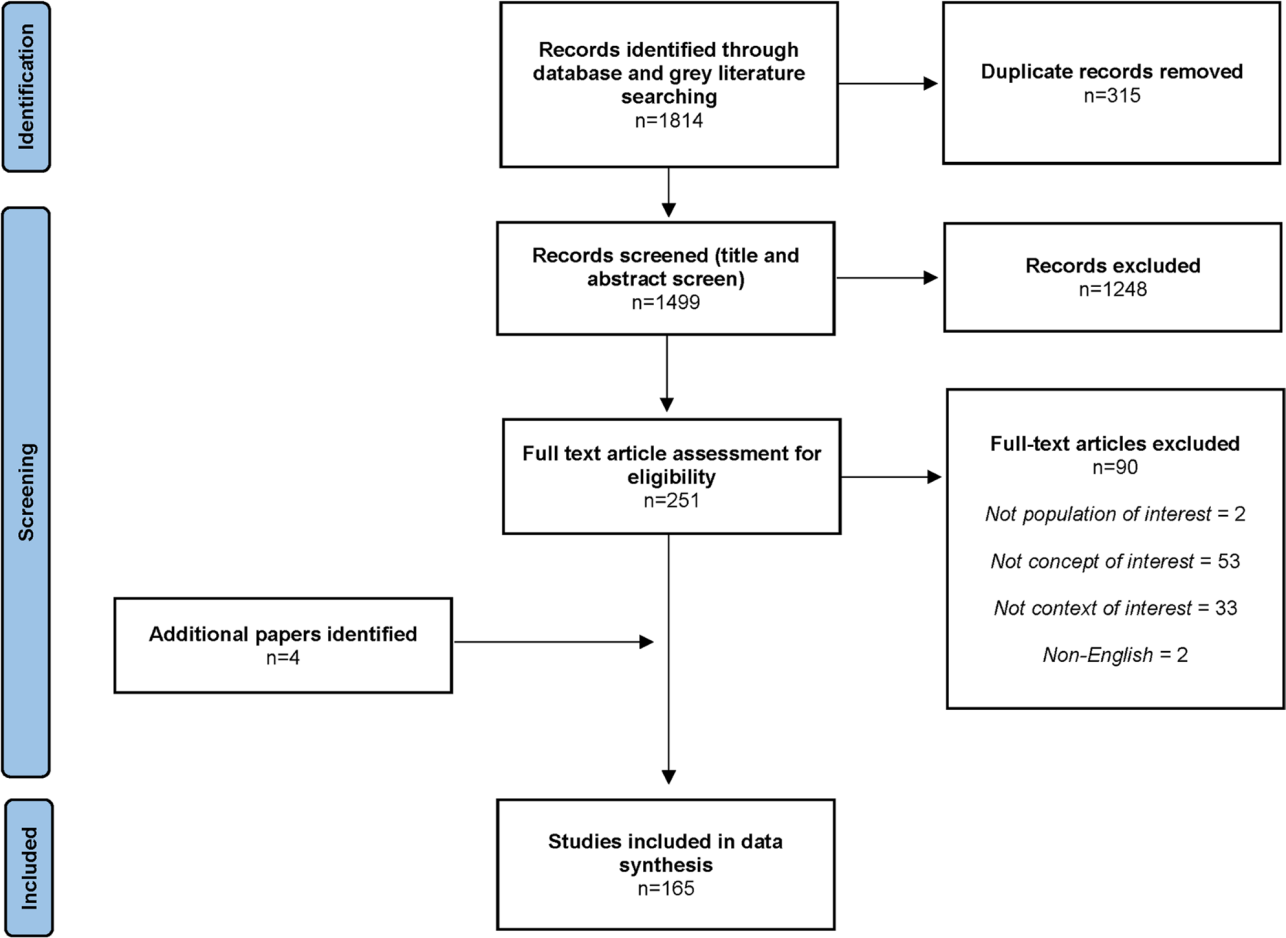
PRISMA flow diagram detailing scoping review process

The search strategy identified 1814 articles (Fig. 1). After removal of duplicates, title and abstract screening, full-text screening, and review of reference and citation lists, 165 articles were included in the final analysis (Supplementary Table 3).

**Fig. 2:**
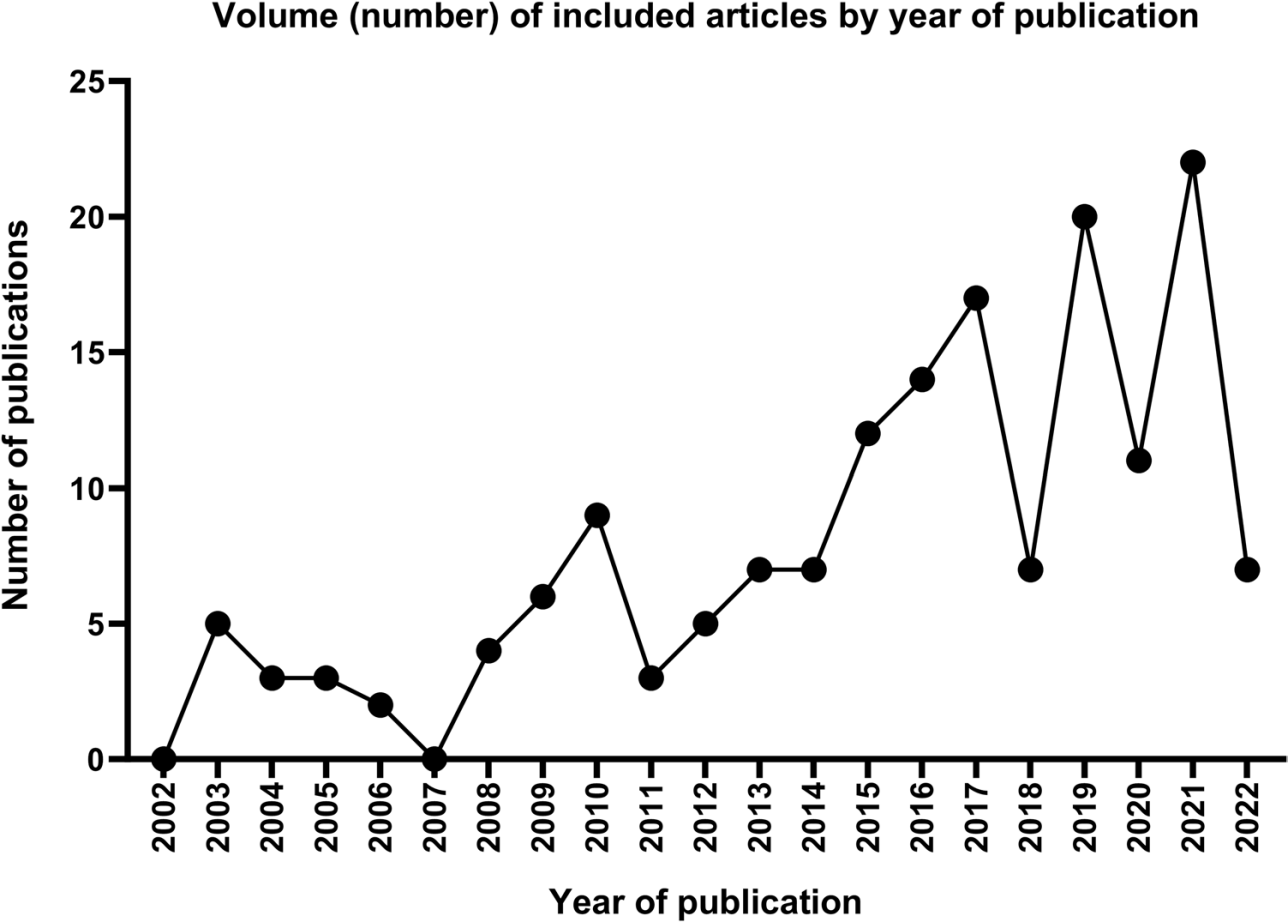
Volume (number) of eligible publications by year of publication

**Fig. 3:**
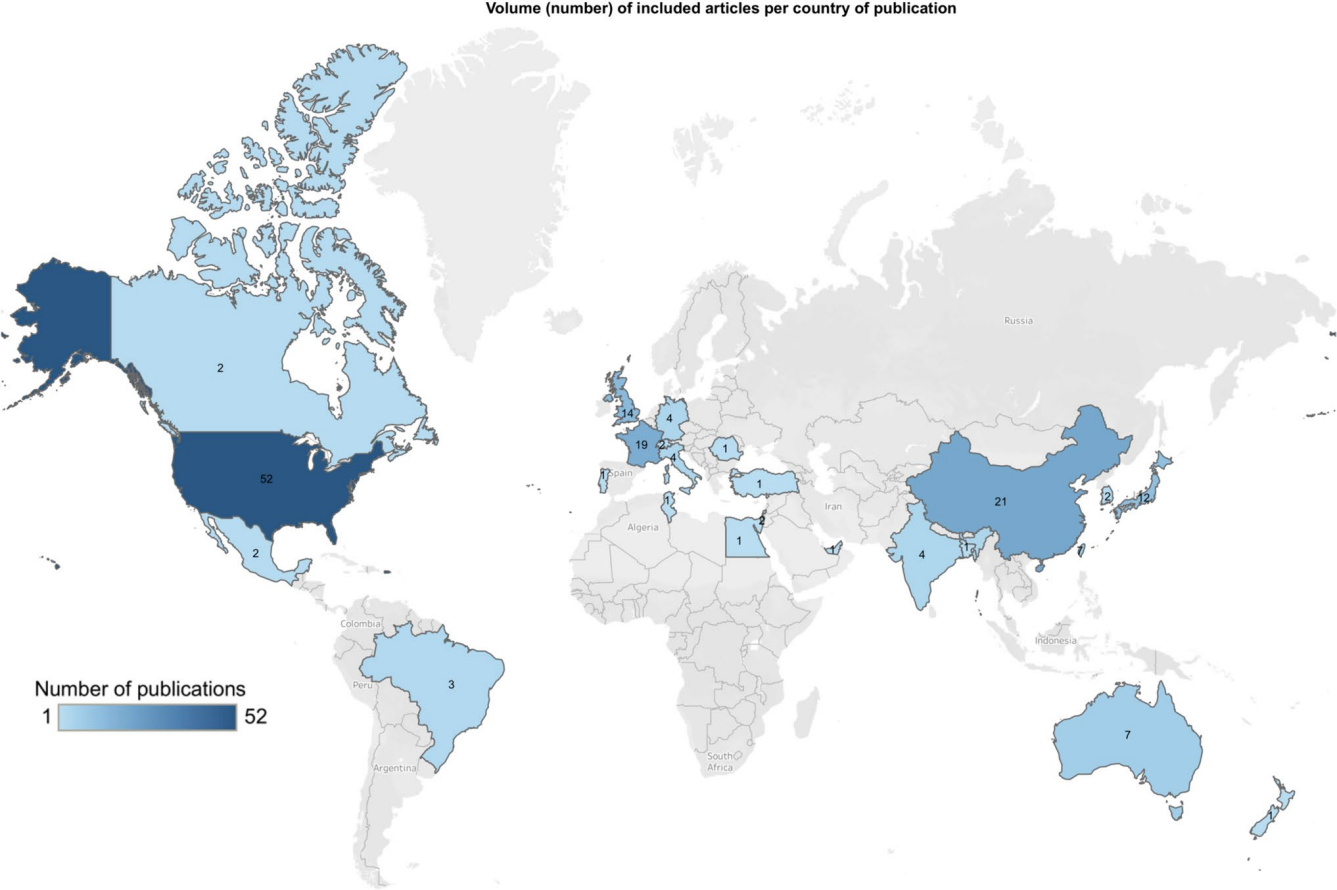
Volume (number) of included articles per country of publication

The most common type of study designs were case reports (51.5%) and retrospective case analyses (29.7%) (Table 1). The year of publication showed a relatively steady increase from 2002 onwards, with most articles being published after 2015 (67.2%) (Fig. 2). Studies originated from all over the globe and included high, middle, and low-income countries. United States was the most common country of origin (31.5%) followed by China (12.7%), France (11.5%) and United Kingdom (8.5%) (Fig. 3). Data pertaining to patient demographics of the study population was obtained from 95 articles for age (122 participants) and 117 articles for gender (974 participants) – most participants were aged between 40–69 years (59.8%) and female (79.6%) (Table 1).

**Table 1:** Patient demographics and design of included studies

	Number (%) patients
**Age**	
< 18 years	8 (6.6)
≥ 18 years	114 (93.4)
**Gender**	
Male	199 (20.4)
Female	775 (79.6)
Other	0 (0)
	Number (%) publications
**Study design**	
Case report	85 (50.6)
Case analysis	
Retrospective	49 (29.2)
Prospective	1 (0.6)
Cohort study	
Retrospective	7 (4.2)
Prospective	3 (1.8)
Pilot study	2 (1.2)
Cost analysis	1 (0.6)
Randomised controlled trial	1 (0.6)
Letter to editor	1 (0.6)
Review	18 (10.7)

### Clinical indications of venous sinus stenting

**Fig. 4:**
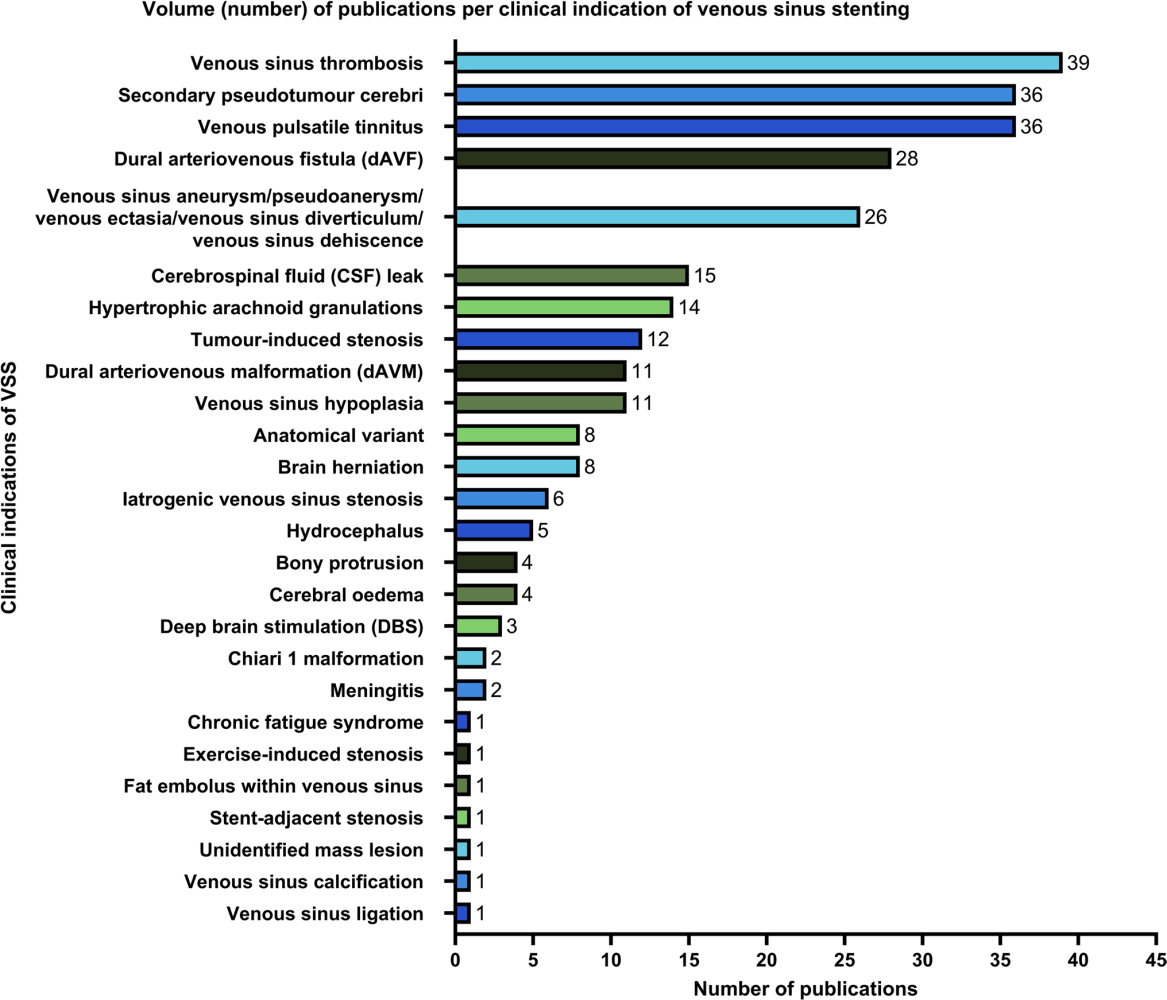
Volume (number) of publications per clinical indication of venous sinus stenting

We identified a total of 27 clinical indications beyond IIH for VSS (Fig. 4). The most commonly reported indications were venous sinus thrombosis (acute or chronic) (23.6%), VPT (21.8%), secondary PTCS (21.8%) and dural arteriovenous fistulae (dAVF) (primary or secondary) (16.9%). Other clinical indications included anatomical variants (congenital or secondary, including high riding jugular bulbs), brain herniation (including encephalocele, pseudomeningocele, meningoencephalocele, and subfalcine herniation), CSF leaks (spontaneous or chronic, sometimes accompanied by spontaneous intracranial hypotension), deep brain stimulation (including for amyotrophic lateral sclerosis, paralysis from spinal cord injury, and muscular dystrophy), acquired hydrocephalus (including traumatic and post-infectious) and tumour-induced stenosis (including meningiomas, paragangliomas, metastatic brain cancer, and a lumbar spine schwannoma). Iatrogenic cases of venous sinus stenosis treated by VSS included venous hypertension post-pituitary adenoma resection, post-acoustic neuroma excision (trans-labyrinthine approach), and post-meningioma debulking, as well as sigmoid sinus laceration post-myringoplasty and refluxed surgical material post-dAVF surgery (Fig. 4). We also investigated the possibility of overlap with overt IIH, and this was only reported in 15 articles (data not shown).

### Technical considerations of venous sinus stenting

**Fig. 5:**
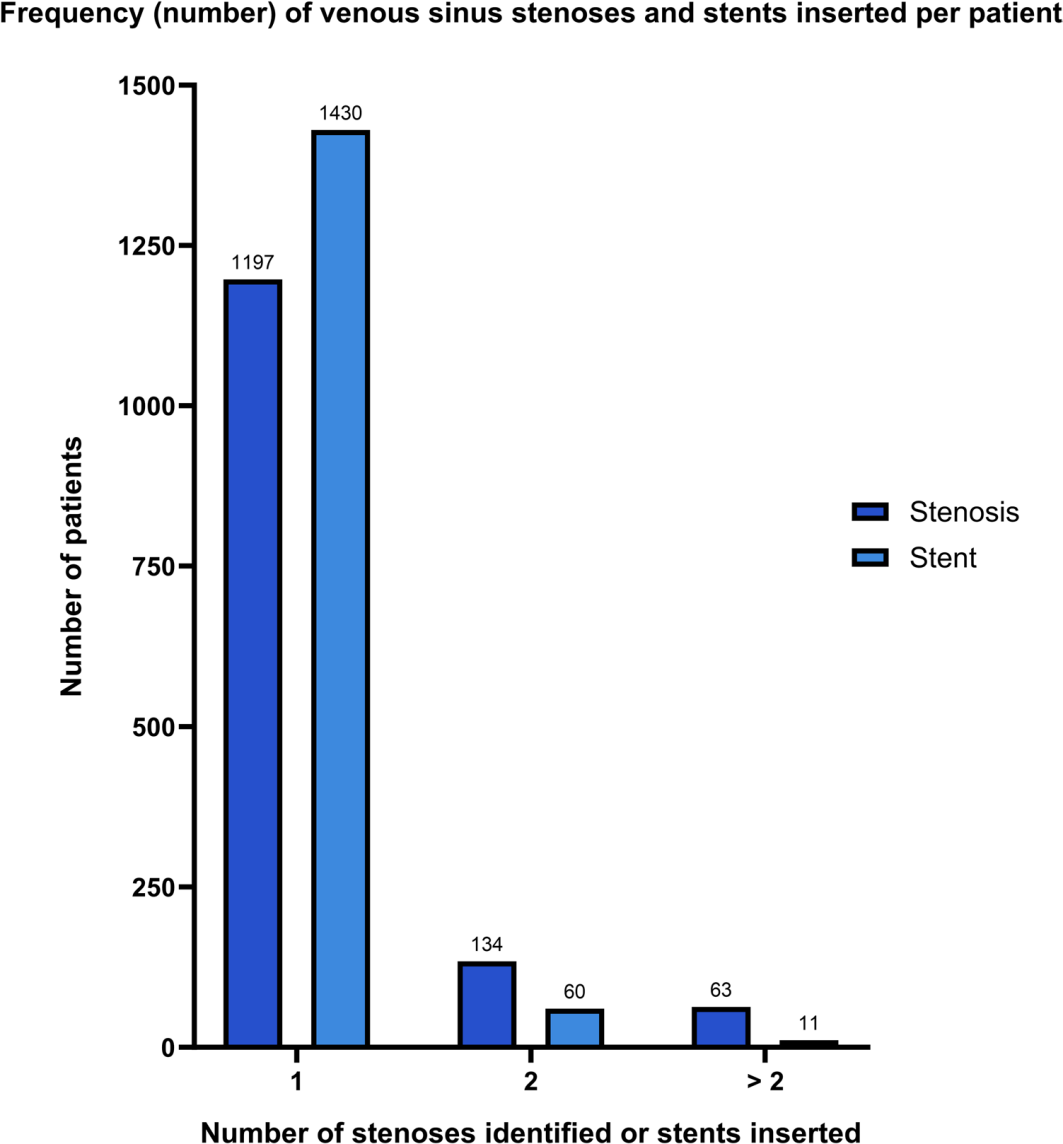
Frequency (number) of venous sinus stenoses and stents inserted per patient

Intracranial location of venous sinus stenosis was reported in 144 articles, of which 118 articles reported lateralisation of the stenosis. Similarly, data on the location of VSS placement was obtained from 137 articles, and data on lateralisation of VSS placement from 89 articles. The most commonly reported location of stenosis and VSS was the transverse sinus, closely followed by the transverse-sigmoid junction, superior sagittal sinus, and sigmoid sinus. Most articles reported right-sided stenoses and right-sided VSS placement, with bilateral stent placement being relatively rare (Table 2). The vast majority of patients had a single stenosis (87.6%) and a single stent inserted (95.3%) (Fig. 5). Measurement of TSPG was reported in 75 articles (45.4%) and ranged from 0 to 60 mmHg, while measurement of proximal venous sinus pressure was reported in an additional 4 articles (2.4%). Intracranial CSF pressure measurement was reported in 58 articles (35.2%) and ranged from 6 to 70 cmH_2_O.

**Table 2:** Frequency and location of venous sinus stenosis and stent placement

	Frequency, n (%)	
	Stenosis	Stent
**Lateralisation**		
Left	166 (38.2)	122 (37.2)
Right	228 (52.5)	174 (53.0)
Bilateral	40 (9.2)	32 (9.8)
**Intracranial location**		
Internal jugular vein	51 (3.1)	43 (3.0)
Inferior sagittal sinus	1 (0.1)	0 (0)
Jugular bulb	4 (0.2)	2 (0.1)
Marginal sinus	9 (0.6)	5 (0.3)
Occipital sinus	4 (0.2)	2 (0.1)
Sigmoid sinus	74 (4.6)	46 (3.2)
Superior sagittal sinus	97 (6.0)	88 (6.1)
Straight sinus	9 (0.6)	9 (0.6)
Superior petrosal sinus	1 (0.1)	1 (0)
Torcula herophili	25 (1.5)	1 (0)
Transverse sinus	714 (44.0)	650 (45.0)
Junction of sigmoid sinus & internal jugular vein	1 (0.1)	5 (0.3)
Junction of transverse sinus & sigmoid sinus	633 (39.0)	592 (40.1)

### Clinical outcomes of venous sinus stenting

One-hundred-and-twenty-eight articles assessed patient outcomes following VSS, with the most common outcome measures being complete resolution of clinical symptoms at last follow-up, neurological recovery, and canalisation or patency on neuroimaging (Table 3). Of these, pulsatile tinnitus and visual impairment showed the greatest resolution at 90.5% and 88.5%, respectively. Most stents remained patent on follow-up neuroimaging (86.3%). Intra- or post-procedural complications of VSS were reported in 34 articles, the most common being in-stent thrombosis (11 articles), intracranial haemorrhage (11 articles), and adjacent (de novo) stenosis (5 articles) (Table 4). Mortality was low at 3 patients (0.7%) and primarily related to intracranial haemorrhage, one of which was in the context of cerebral venous sinus thrombosis.

**Table 3:** Level of symptomatic improvement and reported outcomes following venous sinus stenting

	Number (%) patients	
	Complete resolution	Total
**Clinical symptoms**		
Pulsatile tinnitus	456 (90.5)	504 (100)
Visual impairment	314 (88.5)	355 (100)
Headache	346 (78.5)	441 (100)
Papilloedema	255 (66.1)	386 (100)
Rhinorrhoea	23 (63.9)	36 (100)
	Number (%) patients	
**Level of neurological recovery**		
Complete	371 (85.7)	
Partial	28 (6.5)	
None	31 (7.2)	
Death	3 (0.7)	
	Number (%) patients	
**Level of canalisation or patency on neuroimaging**		
Complete	352 (86.3)	
Partial	13 (3.2)	
None (re-stenosis)	43 (10.5)

**Table 4:** Number of publications reporting venous sinus stenting complications

	Number (%) publications
Allergic reaction	1 (2.1)
Venous sinus fistulation	2 (4.2)
Epistaxis	1 (2.1)
Femoral pseudoaneurysm	1 (2.1)
Groin haematoma	1 (2.1)
Hearing loss	4 (8.3)
Hemiparesis	1 (2.1)
Intracranial haemorrhage (epidural, subdural, subarachnoid, intracerebral)	11 (22.9)
In-stent thrombosis	11 (22.9)
Optic nerve atrophy	2 (4.2)
Stent migration	1 (2.1)
Stent misplacement	1 (2.1)
Stent-adjacent (de novo) stenosis	5 (10.4)
Venous sinus dilation	2 (4.2)
Venous sinus perforation	4 (8.3)
Death	3 (6.3)

## Discussion

We present a scoping review that is, to our knowledge, the first to systematically characterise the practice diversity relating to clinical indications of VSS beyond IIH. We highlight 27 additional indications, mostly obtained from case reports and retrospective case analyses, ranging from intrinsic (e.g. hypertrophic arachnoid granulations) to extrinsic causes of venous sinus stenosis, venous hypertension, and venous outflow obstruction. The identified conditions traverse multiple disciplines within the clinical neurosciences and include diseases that may not have previously utilised endovascular intervention, such as venous sinus thrombosis conventionally managed pharmacologically. Furthermore, it is increasingly recognised that cases diagnosed as IIH have specific underlying aetiologies for their disturbances in intracranial dynamics [12]. For example, 12 primary studies included in this review ostensibly reported the co-existence of IIH in patients who instead fulfilled criteria for secondary PTCS, while patients correctly diagnosed with co-existent IIH from an additional 3 articles were excluded entirely [22, 24]. Similar ambiguity was noted in the context of CSF leaks, as IIH patients can present with encephaloceles resulting in spontaneous rhinorrhoea, though targeted analysis of the largest case series included in this review (data not shown) revealed absence of IIH symptomology (such as elevated CSF pressure or clinical signs of raised ICP) & high primary resolution rates in our cohort, which is atypical for overt IIH-induced CSF leaks [31]. VPT, a common consequence of IIH, also required additional scrutiny, but multifactorial aetiology and absence of IIH characteristics in these cases meant the authors were confident that VPT served as its own distinct indication for VSS [12, 23]. Further targeted examination of these interdisciplinary conditions is necessary, however, to assess global clinical practice and determine how different service providers may be employing VSS in different ways. Given the growing focus in the field on the neuropsychiatric impact of intracranial hypertension, as well as the increased interest in this procedure amongst neuroradiological circles, we note that cross-specialty collaboration is important for future innovation in this space. To this end, this review hopefully demonstrates that while VSS is being utilised in a variety of clinical settings, much work is indicated to optimise the safety of service provision and improve patient outcomes.

There was a considerable degree of clinical heterogeneity noted within the reviewed literature, particularly in the context of assessing symptom severity and post-intervention outcomes. For reporting purposes, clinical symptom resolution was dichotomised as complete or incomplete, since increased granularity was not possible due to notable differences in the methodological approaches used between studies. We also identified the type and extent of post-procedural complications, but an assessment of how often these occur and whether this degree of risk is warranted was not possible with the identified evidence. The most commonly reported symptoms in this review (pulsatile tinnitus, visual impairment, and headache) are typical of venous outflow obstruction, rather than the specific underlying causes. Despite these symptoms showing the greatest clinical resolution, in practice they can be quite challenging to quantitatively evaluate, as are the more subtle aspects of intracranial hypertension, such as the impact on cognition, affect, and behaviour [3, 5, 13, 37]. To this end, the development of robust core outcome measures with multi-stakeholder consensus is necessary to support standardised stenting service practice [9]. Following on from this, more robust research beyond case reports and retrospective case analyses is essential to strengthen the evidence base of the long-term efficacy and safety of VSS. This will ideally take the form of randomised controlled trials (RCTs), whose trial design and post-RCT implementation will be informed by appropriate registries or observational cohort studies for long-term pooled sample surveillance, and followed by systematic review and meta-analysis. However, the relative rarity of certain clinical indications identified by this review may make randomisation challenging, particularly if VSS is combined with other interventional modalities, such as the dual stent-electrode (“stentrode”) system for deep brain stimulation [6, 27, 28]. Therefore, it is paramount to first optimise VSS practice for the core indications (i.e. venous sinus pathology) before exploring more innovative applications of the technique that may merit more tailored evaluation. Nonetheless, evidence continues to grow in support of VSS for IIH, the most common application of VSS, with multiple clinical trials underway or complete [8, 29, 30].

A significant burden on subjective clinician judgement for deciding the suitability of VSS exists and contributes extensively to practice variability between service providers. Whilst TSPG measurement is considered gold standard, many clinicians presently rely on static image assessment for determining the severity of venous sinus abnormalities, which provides no information on the nature of stenoses, or potential causal mechanisms. Fixed anatomical stenoses differ in their pathophysiology from physiological disorders of intracranial dynamics, such as IIH, where there is an increased risk of stent-adjacent stenosis secondary to pathologically flaccid and collapsible venous anatomy, as seen with coronary and peripheral vasculature [19]. We show that a moderate number of papers utilised a dynamic measure of TSPG to supplement decision-making when determining the suitability of VSS. However, the significance of this is not fully understood, and recent reports in IIH have reported a therapeutic benefit even with low pressure gradient stenoses [17]. Assessing TSPGs through the utilisation of novel imaging techniques, particularly dynamic MRI and angiography, may be of particular benefit here to improve objectivity [7, 26, 34]. We also note considerable practice variability in terms of the choice of intracranial stenting location, choice of post-procedural anti-platelet regime, and the management of intervention-refractory cases, where some service providers may choose to repeatedly re-stent despite potentially diminishing returns, while others may resort to other diagnostic tests to supplement their clinical judgement.

The fields of endovascular neurosurgery and interventional neuroradiology are rapidly evolving, and recent advancements emphasise the timeliness of this scoping review. Winkler et al. (2022) recently demonstrated a trans-venous endovascular biopsy approach for molecular profiling of brain vascular malformations, while Lylyk et al. (2021) described the first endovascular trans-dural approach for CSF shunting in hydrocephalus [21, 36]. As the neurovascular community starts to shift focus from arterial pathology to venous disease, identifying future research avenues becomes of paramount importance to generate impactful changes to the target patient populations. This scoping review is therefore critical in raising awareness amongst service providers that VSS can be employed in their practice for diverse clinical indications beyond IIH. While this review offers an overview of the field two decades after the introduction of VSS, further refinement is necessary to support the wider adoption of VSS in modern clinical practice [15].

### Methodological considerations

A scoping review was the most appropriate methodology for our research question due to the anticipated heterogeneity of included studies and the breadth of the research topic. However, as a scoping review, the study goal was to chart currently available literature, rather than to give recommendations, and therefore a formal assessment of the methodological quality of studies and risk of bias was not performed. Furthermore, many diagnostic indications reported in individual studies can have considerable overlap but attempts to group these potentially linked aetiologies is beyond the scope of this review. Additionally, this study was limited to only considering existing published literature, though the authors postulate that there are likely unpublished or non-English projects that further demonstrate the utility of VSS beyond IIH. Nonetheless, scoping reviews provide a rigorous framework to examine the extent, range, and nature of research activity, as well as summarise and disseminate research findings.

## Conclusion

Our review provides a detailed overview of the clinical indications of VSS beyond IIH and increases the awareness of VSS as a primary or adjunct therapy for a variety of neurovascular pathologies and diseases encountered in various branches of the clinical neurosciences. We note the extensive impact of heterogeneity in VSS literature and practice, as well as the importance of developing core outcome measures, improved diagnostic accuracy, and greater primary research data looking at long-term efficacy of VSS in the identified clinical indications, where possible. Our findings will help inform endovascular neurosurgeons, interventional neuroradiologists, and clinical commissioners, as well as help in the design of future research on the topic and standardise the delivery of VSS globally.

## Supplementary Information

Below is the link to the electronic supplementary material.

Supplementary File 1: Preferred Reporting Items for Systematic reviews and Meta-Analyses extension for Scoping Reviews (PRISMA-ScR) Checklist (PDF 245 KB)

Supplementary File 2: Summary of MEDLINE (Ovid) Search Strategy (PDF 95 KB)

Supplementary File 3: List of Included Articles (PDF 174 KB)

## Data Availability

No datasets were generated or analysed during the current study.
