# Supplementary material for: Clinical indications and patient outcomes of intracranial venous sinus stenting beyond overt idiopathic intracranial hypertension: a scoping review
Source: Acta Neurochir (Wien). 2025 Apr 25;167(1):122. doi: 10.1007/s00701-025-06514-7 (PMC12031942; doi:10.1007/s00701-025-06514-7)
Supplement: Supplementary file 2 — Summary of MEDLINE (Ovid) Search Strategy (PDF 95 KB) [file 701_2025_6514_MOESM2_ESM.pdf]

Summary of MEDLINE (Ovid) search strategy		
<u>Search</u>	<u>Query</u>	<u>Records retrieved</u>
#1	veno* OR cerebral OR intracranial OR cranial OR dural OR transverse OR cavernous OR petrosal OR sigmoid* OR basilar OR sagittal OR straight OR occipital OR intercavernous	1178616
#2	sinus*	181612
#3	stent*	113217
#4	(#1 ADJ5 #2 ADJ5 #3).mp.	257
Limited to English language, human studies, and year (2002 – Current)		234
