# Supplementary material for: Clinical indications and patient outcomes of intracranial venous sinus stenting beyond overt idiopathic intracranial hypertension: a scoping review
Source: Acta Neurochir (Wien). 2025 Apr 25;167(1):122. doi: 10.1007/s00701-025-06514-7 (PMC12031942; doi:10.1007/s00701-025-06514-7)
Supplement: Supplementary file 3 — List of Included Articles (PDF 174 KB) [file 701_2025_6514_MOESM3_ESM.pdf]

List of included articles (in alphabetical order):

1. Aaron S, Arthur A, Prabakhar AT, *et al*. Spectrum of Visual Impairment in Cerebral Venous Thrombosis: Importance of Tailoring Therapies Based on Pathophysiology. *Ann Indian Acad Neurol*. 2017;20(3):294-301. doi:10.4103/aian.AIAN_11_17
2. Adachi H, Mineharu Y, Ishikawa T, *et al*. Stenting for acute cerebral venous sinus thrombosis in the superior sagittal sinus. *Interv Neuroradiol*. 2015;21(6):719-723. doi:10.1177/1591019915609120
3. Ahmed RM, Zmudzki F, Parker GD, *et al*. Transverse sinus stenting for pseudotumor cerebri: a cost comparison with CSF shunting. *AJNR Am J Neuroradiol*. 2014;35(5):952-958. doi:10.3174/ajnr.A3806
4. Almadidy Z, Brunozzi D, Nelson J, *et al*. Intracranial venous sinus stenosis: hemodynamic assessment with two-dimensional parametric parenchymal blood flow software on digital subtraction angiography. *J Neurointerv Surg*. 2020;12(3):311-314. doi:10.1136/neurintsurg-2019-015582
5. Altieri C, Caldas JMP, Ferraz FM, *et al*. Angioplasty with auto-expandable stent as treatment for symptomatic chronic cerebral venous thrombosis: A report of 2 cases. *J Neurol Sci*. 2017;381:392-393. doi:10.1016/j.jns.2017.08.3324
6. Bai C, Chen J, Wu X, *et al*. Perioperative mannitol intensive use may avoid the early complication of cerebral venous sinus stenting. *Ann Transl Med*. 2020;8(11):672. doi:10.21037/atm-20-3021
7. Bai C, Chen Z, Wu X, *et al*. Safety and efficacy comparison between OACs plus single antiplatelet and dual antiplatelet therapy in patients with cerebral venous sinus stenosis poststenting. *BMC Neurol*. 2022;22(1):209. doi:10.1186/s12883-022-02731-0
8. Baomin L, Yongbing S, Xiangyu C. Angioplasty and stenting for intractable pulsatile tinnitus caused by dural venous sinus stenosis: a case series report. *Otol Neurotol*. 2014;35(2):366-370. doi:10.1097/MAO.0b013e3182990d52
9. Baomin L, Xiangyu C, Xinfeng L, *et al*. Interventionai diagnosis and treatment of vasculogeneic pulsatile tinnitus. *J Otol*. 2014:9(1):7-15. doi:10.1016/S1672-2930(14)50002-5
10. Bedarida V, Labeyrie MA, Eliezer M, *et al*. Association of spontaneous cerebrospinal fluid rhinorrhea with transverse venous sinus stenosis: a retrospective matched case-control study. *Int Forum Allergy Rhinol*. 2020;10(12):1295-1299. doi:10.1002/alr.22660
11. Beer-Furlan A, Munich SA, Chen M. Augmenting superior sagittal sinus functionality. Commentary: Motor neuroprosthesis implanted with neurointerventional surgery improves capacity for activities of daily living tasks in severe paralysis-first in human experience. *J Neurointerv Surg*. 2021;13(2):100-101. doi:10.1136/neurintsurg-2020-017074
12. Beiruti K, Shadi J, Shahien R, *et al*. Critical management of cerebral venous sinus thrombosis (CVST) concomitant with high intracranial pressure (ICP). *J Neurol Sci*. 2017;381:398. doi:10.1016/j.jns.2017.08.3338
13. Benveniste RJ, Patel AB, Post KD. Management of cerebral venous sinus thrombosis. *Neurosurg Q*. 2004:14(1):27-35.

14. Bhogal P, AlMatter M, Aguilar M, *et al*. Cerebral Venous Sinus Thrombosis : Endovascular Treatment with Rheolysis and Aspiration thrombectomy. *Clin Neuroradiol*. 2017;27(2):235-240. doi:10.1007/s00062-016-0540-1
15. Buell T, Raper D, Pomeraniec J, *et al*. A Pilot Study on the Safety and Efficacy of Stenting Superior Sagittal Sinus Stenosis and a New Angiographic Naming System. *J Neurosurg*. 2017;126(4):A1396-A1396.
16. Buell TJ, Raper DMS, Ding D, *et al*. Concurrent Venous Stenting of the Transverse and Occipito-Marginal Sinuses: An Analogy with Parallel Hemodynamic Circuits. *J Neurosci Rural Pract*. 2019;10(2):334-338. doi:10.4103/jnrp.jnrp_259_18
17. Burger BM, Chavis PS, Purvin V. A weed by any other name. *Surv Ophthalmol*. 2013;58(2):176-183. doi:10.1016/j.survophthal.2012.02.005
18. Cabral de Andrade G, Lesczynsky A, Clímaco VM, *et al*. Cerebral venous sinuses thrombosis in both transverse sinus and torcula: Multistep endovascular treatment and stenting. *Interv Neuroradiol*. 2017;23(1):84-89. doi:10.1177/1591019916674917
19. Cayli E, Oguz S, Dinc H. Mechanical Extraction of a Refluxed Onyx Piece from the Sigmoid Sinus Using a Solitaire AB Stent for Treating a Dural Arteriovenous Fistula: technical report. *Eurasian J Med*. 2017;49(3):224-225. doi:10.5152/eurasianjmed.2017.17260
20. Chahbazian K, Théaudin M, Lehmann P, *et al*. Reversible pseudo-Creutzfeldt-Jakob syndrome related to cerebral dural arteriovenous fistula. *J Am Geriatr Soc*. 2014;62(10):2024-2026. doi:10.1111/jgs.13047
21. Chausson N, Bocquet J, Aveillan M, *et al*. Intracranial hypertension caused by a meningioma compressing the transverse sinus. *J Clin Neurosci*. 2010;17(12):1589-1592. doi:10.1016/j.jocn.2010.03.039
22. Chen C, Wang Q, Li X, *et al*. Stent retriever thrombectomy combined with local thrombolytic therapy for cerebral venous sinus thrombosis: A case report. *Exp Ther Med*. 2017;14(5):3961-3970. doi:10.3892/etm.2017.5043 [published correction appears in Exp Ther Med. 2018 Mar;15(3):2694]
23. Chen KW, Lin YH, Lee CW. Acute Posttraumatic Cerebral Venous Sinus Thrombosis-Induced Malignant Increased Intracranial Pressure Treated with Endovascular Dural Sinus Thrombectomy and Stenting. *World Neurosurg*. 2019;128:393-397. doi:10.1016/j.wneu.2019.05.105
24. Choi BJ, Lee TH, Kim CW, *et al*. Reconstructive treatment using a stent graft for a dural arteriovenous fistula of the transverse sinus in the case of hypoplasia of the contralateral venous sinuses: technical case report. *Neurosurgery*. 2009;65(5):E994-E996. doi:10.1227/01.NEU.0000351772.45417.92
25. Chung CY, John S, Luciano MG, *et al*. Reduction in Syrinx Size and Severity After Venous Sinus Stenting in a Patient With Pseudotumor Cerebri and Chiari Malformation: Technical Case Report. *Oper Neurosurg (Hagerstown)*. 2016;12(2):E197-E201. doi:10.1227/NEU.0000000000001211

26. Comay Y, Zvenigorodsky V, Borodetsky V, *et al*. Cerebral Venous Stenting for Pulsatile Tinnitus After Unsuccessful Jugular Ligation: Case Report and Literature Review. *Ear Nose Throat J*. 2022;101(3):153-157. doi:10.1177/0145561320946155
27. Cortese J, Eliezer M, Guédon A, *et al*. Pulsatile Tinnitus Due to Stenosis of the Marginal Sinus: Diagnosis and Endovascular Treatment. *AJNR Am J Neuroradiol*. 2021;42(12):2194-2198. doi:10.3174/ajnr.A7325
28. Costa M, Tataryn Z, Alobaid A, *et al*. Robotically-assisted neuro-endovascular procedures: Single-Center Experience and a Review of the Literature. *Interv Neuroradiol*. 2022;15910199221082475. doi:10.1177/15910199221082475
29. Craven C, Toma AK, Khan AA, *et al*. The role of ICP monitoring in patients with persistent cerebrospinal fluid leak following spinal surgery: a case series. *Acta Neurochir (Wien)*. 2016;158(9):1813-1819. doi:10.1007/s00701-016-2882-5
30. Crawford A, McGrath NM. Hearing loss after lumbar puncture. *J Clin Neurosci*. 2010;17(1):149-152. doi:10.1016/j.jocn.2009.04.006
31. Cuellar H, Maiti T, Patra DP, *et al*. Endovascular Treatment of Pulsatile Tinnitus by Sigmoid Sinus Aneurysm: Technical Note and Review of the Literature. *World Neurosurg*. 2018;113:238-243. doi:10.1016/j.wneu.2018.02.087
32. Daggubati LC, Liu KC. Intracranial Venous Sinus Stenting: A Review of Idiopathic Intracranial Hypertension and Expanding Indications. *Cureus*. 2019;11(2):e4008. doi:10.7759/cureus.4008
33. David E, Mankad K. Brain Imaging in Pediatric Pseudotumor Cerebri Syndrome. *J Pediatr Neurol*. 2015;13(01):049-053. doi:10.1055/s-0035-1555155
34. Ding D, Starke RM, Durst CR, *et al*. Venous stenting with concurrent intracranial pressure monitoring for the treatment of pseudotumor cerebri. *Neurosurg Focus*. 2014;37(1 Suppl):1. doi:10.3171/2014.V2.FOCUS14162
35. Ding D, Chen CJ, Starke RM, *et al*. Rapid recovery of bilateral abducens nerve palsies after venous sinus stenting for idiopathic intracranial hypertension. *J Neurol Sci*. 2015;357(1-2):335-337. doi:10.1016/j.jns.2015.07.047
36. Ding J, Guan J, Ji X, *et al*. Cerebral Venous Sinus Stenosis may Cause Intracranial Arterial Hypoperfusion. *Clin Neuroradiol*. 2020;30(2):409-411. doi:10.1007/s00062-019-00833-w
37. Drocton GT, Copelan A, Eisenmenger L, *et al*. Venous sinus stenting as a treatment approach in patients with idiopathic intracranial hypertension and encephaloceles. *Interv Neuroradiol*. 2021;27(1):129-136. doi:10.1177/1591019920956860
38. Du Z, Liu X, Cao X, *et al*. Pearls & Oy-sters: Retrievable and awake: A case report of solitaire stent employment for venous pulsatile tinnitus. *Neurology*. 2017;88(24):e245-e248. doi:10.1212/WNL.0000000000004032

39. Dutmers J, Soule E, Bertran MA, *et al*. Side-by-Side Stenting Repair of a Traumatic Pseudoaneurysm at a Venous Confluence. *Vasc Endovascular Surg*. 2020;54(5):406-412. doi:10.1177/1538574420921014
40. El Mekabaty A, Pearl MS, Moghekar A, *et al*. Mid-term assessment of transverse sinus stent patency in 104 patients treated for intracranial hypertension secondary to dural sinus stenosis. *J Neurointerv Surg*. 2021;13(2):182-186. doi:10.1136/neurintsurg-2020-015949
41. Entezami P, Gooch MR, Dalfino J. Endovascular stenting of the superior sagittal sinus to alleviate venous compression caused by a parasagittal meningioma. *BMJ Case Rep*. 2019;12(4):e227935. doi:10.1136/bcr-2018-227935
42. Esfahani DR, Stevenson M, Moss HE, *et al*. Quantitative Magnetic Resonance Venography is Correlated With Intravenous Pressures Before and After Venous Sinus Stenting: Implications for Treatment and Monitoring. *Neurosurgery*. 2015;77(2):254-260. doi:10.1227/NEU.0000000000000771
43. Fargen KM. Idiopathic intracranial hypertension is not idiopathic: proposal for a new nomenclature and patient classification. *J Neurointerv Surg*. 2020;12(2):110-114. doi:10.1136/neurintsurg-2019-015498
44. Fargen KM. Venous stenting for idiopathic intracranial hypertension: lessons learned from a high-volume practice. *J Neurointerv Surg*. 2022;14(6):528-532. doi:10.1136/neurintsurg-2021-018184
45. Fargen KM, Velat GJ, Lewis SB, *et al*. Concomitant intracranial pressure monitoring during venous sinus stenting for intracranial hypertension secondary to venous sinus stenosis. *J Neurointerv Surg*. 2013;5(4):e22. doi:10.1136/neurintsurg-2012-010371
46. Fargen KM, Spiotta AM, Hyer M, *et al*. Comparison of venous sinus manometry gradients obtained while awake and under general anesthesia before venous sinus stenting. *J Neurointerv Surg*. 2017;9(10):990-993. doi:10.1136/neurintsurg-2016-012608
47. Farid M, Alawamry A, Zaitoun MMA, *et al*. Relentless pulsatile tinnitus secondary to dural sinovenous stenosis: is endovascular sinus stenting the answer?. *Clin Radiol*. 2021;76(7):526-531. doi:10.1016/j.crad.2021.02.022
48. Farina R, Foti PV, Pennisi I, *et al*. Stylo-Jugular Venous Compression Syndrome: Lessons Based on a Case Report. *Am J Case Rep*. 2021;22:e932035. doi:10.12659/AJCR.932035
49. Fiani B, Kondilis A, Doan T, *et al*. Venous sinus stenting for intractable pulsatile tinnitus: A review of indications and outcomes. *Surg Neurol Int*. 2021;12:81. doi:10.25259/SNI_1_2021
50. Formaglio M, Catenoix H, Tahon F, *et al*. Stenting of a cerebral venous thrombosis. *J Neuroradiol*. 2010;37(3):182-184. doi:10.1016/j.neurad.2009.08.001
51. Fukumoto S, Ueda T, Igase K, *et al*. Stenting procedure for sinus stenosis with transverse-sigmoid dural arteriovenous fistulas. A case report. *Interv Neuroradiol*. 2006;12(Suppl 1):178-184. doi:10.1177/15910199060120S131

52. Ganesan D, Higgins JN, Harrower T, *et al*. Stent placement for management of a small parasagittal meningioma. Technical note. J Neurosurg. 2008;108(2):377-381. doi:10.3171/JNS/2008/108/2/0377
53. Ghali GZ, Ghali MG, Ghali EZ, *et al*. Intracranial Venous Hypertension in Craniosynostosis: Mechanistic Underpinnings and Therapeutic Implications. World Neurosurg. 2019;127:549-558. doi:10.1016/j.wneu.2018.07.260
54. Gross BA, Albuquerque FC, Moon K, *et al*. New frontiers in venous sinus stenting: Illustrative cases. *J Clin Neurosci*. 2016;33:241-244. doi:10.1016/j.jocn.2016.05.032
55. Guédon A, Labeyrie MA, Civelli V, *et al*. Navigability of a long sheath in the lateral dural sinuses facilitated by the pilot balloon technique: technical note. *Neuroradiology*. 2021;63(12):2149-2151. doi:10.1007/s00234-021-02776-x
56. Guo WY, Lee CJ, Lin CJ, *et al*. Quantifying the Cerebral Hemodynamics of Dural Arteriovenous Fistula in Transverse Sigmoid Sinus Complicated by Sinus Stenosis: A Retrospective Cohort Study. *AJNR Am J Neuroradiol*. 2017;38(1):132-138. doi:10.3174/ajnr.A4960
57. Gupta A, Periakaruppan A. Intracranial dural arteriovenous fistulas: A Review. *Indian J Radiol Imaging*. 2009;19(1):43-48. doi:10.4103/0971-3026.45344
58. Gupta G, Rallo MS, Goldrich DY, *et al*. Management of Jugular Bulb Stenosis in Pediatric Vein of Galen Malformation: A Novel Management Paradigm. *Pediatr Neurosurg*. 2021;56(6):584-590. doi:10.1159/000517653
59. Gutierrez A, Do HM, Marks MP. Alteration in the venous drainage of a dural arteriovenous fistula following angioplasty. *AJNR Am J Neuroradiol*. 2004;25(6):1086-1088.
60. Han Y, Yang Q, Yang Z, *et al*. Computational Fluid Dynamics Simulation of Hemodynamic Alterations in Sigmoid Sinus Diverticulum and Ipsilateral Upstream Sinus Stenosis After Stent Implantation in Patients with Pulsatile Tinnitus. *World Neurosurg*. 2017;106:308-314. doi:10.1016/j.wneu.2017.06.168
61. Harsha KJ. Successful Endovascular Neurosurgical Practice in Resource-poor Exclusive Rural Neuro-hospital Setup. *J Neurosci Rural Pract*. 2016;7(Suppl 1):S13-S17. doi:10.4103/0976-3147.196459
62. Hartmann AJPW, Latting MW, Lee MS, *et al*. Papilloedema from Dural Venous Sinus Compression by Meningiomas. *Neuroophthalmology*. 2018;43(3):171-179. doi:10.1080/01658107.2018.1524499
63. Higgins JN, Axon PR, Macfarlane R. Spontaneous Intracranial Hypotension Complicated by Subdural Effusions Treated by Surgical Relief of Cranial Venous Outflow Obstruction. *J Neurol Surg Rep*. 2020;81(4):e59-e65. doi:10.1055/s-0040-1722268
64. Higgins JN, Pickard JD. Intractable headache after excision of an acoustic neuroma treated by stent revascularisation of the sigmoid sinus. *Br J Neurosurg*. 2013;27(6):819-821. doi:10.3109/02688697.2013.791665

65. Higgins JN, Kirkpatrick PJ. Stenting venous outflow gives symptomatic improvement in a patient with an inoperable brainstem arteriovenous malformation. *Br J Neurosurg*. 2013;27(5):698-700. doi:10.3109/02688697.2013.795524
66. Higgins JN, Burnet NG, Schwindack CF, *et al*. Severe brain edema caused by a meningioma obstructing cerebral venous outflow and treated with venous sinus stenting. Case report. *J Neurosurg*. 2008;108(2):372-376. doi:10.3171/JNS/2008/108/2/0372
67. Higgins JN, Garnett MR, Pickard JD, *et al*. An Evaluation of Styloidectomy as an Adjunct or Alternative to Jugular Stenting in Idiopathic Intracranial Hypertension and Disturbances of Cranial Venous Outflow. *J Neurol Surg B Skull Base*. 2017;78(2):158-163. doi:10.1055/s-0036-1594238
68. Higgins JN, Macfarlane R, Axon PR, *et al*. Headache, Cerebrospinal Fluid Leaks, and Pseudomeningoceles after Resection of Vestibular Schwannomas: Efficacy of Venous Sinus Stenting Suggests Cranial Venous Outflow Compromise as a Unifying Pathophysiological Mechanism. *J Neurol Surg B Skull Base*. 2019;80(6):640-647. doi:10.1055/s-0039-1677706
69. Higgins JN, Pickard J, Lever A. Borderline Intracranial Hypertension Manifesting as Chronic Fatigue Syndrome Treated by Venous Sinus Stenting. *J Neurol Surg Rep*. 2015;76(2):e244-e247. doi:10.1055/s-0035-1564060
70. Higgins JN, Trivedi R, Greenwood R, *et al*. Brain Slump Caused by Jugular Venous Stenoses Treated by Stenting: A Hypothesis to Link Spontaneous Intracranial Hypotension with Idiopathic Intracranial Hypertension. *J Neurol Surg Rep*. 2015;76(1):e188-e193. doi:10.1055/s-0035-1555015
71. Hirata E, Higashi T, Iwamuro Y, *et al*. Angioplasty and stent deployment in acute sinus thrombosis following endovascular treatment of dural arteriovenous fistulae. *J Clin Neurosci*. 2009;16(5):725-727. doi:10.1016/j.jocn.2008.07.071
72. Hitier M, Barbier C, Marie-Aude T, *et al*. New treatment of vertigo caused by jugular bulb abnormalities. *Surg Innov*. 2014;21(4):365-371. doi:10.1177/1553350613505918
73. Higgins JN, Owler BK, Cousins C, *et al*. Venous sinus stenting for refractory benign intracranial hypertension. *Lancet*. 2002;359(9302):228-230. doi:10.1016/S0140-6736(02)07440-8
74. Honarmand AR, Hurley MC, Ansari SA, *et al*. Focal stenosis of the sigmoid sinus causing intracranial venous hypertension: Case report, endovascular management, and review of the literature. *Interv Neuroradiol*. 2016;22(2):240-245. doi:10.1177/1591019915622160
75. Hu W, Wang C, Wu Q, *et al*. Intracranial hypertension due to spinal cord tumor misdiagnosed as pseudotumor cerebri syndrome: case report. *BMC Neurol*. 2020;20(1):420. doi:10.1186/s12883-020-02000-y
76. Hueng DY, Ma HI, Chen CM. Cushing's disease and dural invasion. *J Neurosurg*. 2012;117(1):189-191. doi:10.3171/2012.2.JNS111890
77. Iencean SM, Poeata I, Iencean AS, *et al*. Cerebral venous etiology of intracranial hypertension and differentiation from idiopathic intracranial hypertension. *Kaohsiung J Med Sci*. 2015;31(3):156-162. doi:10.1016/j.kjms.2014.12.007

78. Ionescu EC, Coudert A, Reynard P, *et al*. Stenting the Superior Petrosal Sinus in a Patient With Symptomatic Superior Semicircular Canal Dehiscence. *Front Neurol*. 2018;9:689. doi:10.3389/fneur.2018.00689

79. Iyer RR, Solomon D, Moghekar A, *et al*. Venous Sinus Stenting in the Management of Patients with Intracranial Hypertension Manifesting with Skull Base Cerebrospinal Fluid Leaks. *World Neurosurg*. 2017;106:103-112. doi:10.1016/j.wneu.2017.06.087

80. Jain VK, Singh V, Kannaujia V, *et al*. Papilledema revisiting after sinus angioplasty of chronic cerebral venous sinus thrombosis. *Indian J Ophthalmol*. 2018;66(5):714-717. doi:10.4103/ijo.IJO_930_17

81. Jaulent P, Vignot E, Chapurlat R. Fibrous dysplasia of occipital bone revealed by acute intracranial hypertension. *Osteoporos Int*. 2019;30(3):691-693. doi:10.1007/s00198-018-4737-9

82. Ji X, Chen J. Stenting angioplasty in the treatment of venous sinus stenoses : Clinical and angiographic outcome in 34 patients. *Stroke*. 2011;42(3):e237, doi:10.1161/STR.0b013e3182074d9b

83. Jia M, Guo ZN, Jin H, *et al*. Venous sinus stenting improves cerebral autoregulation in a patient with venous sinus stenosis: a case report. *BMC Neurol*. 2020;20(1):9. doi:10.1186/s12883-019-1595-9

84. Jovin T, Aghaebrahim A. Endovascular Management of Diseases in Relation to Otolaryngology. *Otolaryngol Clin North Am*. 2016;49(3):841-862. doi:10.1016/j.otc.2016.03.005

85. Kassis S, Lugovsky A, Msaddi AK, *et al*. Endovascular treatment of transverse sinus aneurysm presenting with occipital headache. *J Neuroradiol*. 2011;38(2):129-130. doi:10.1016/j.neurad.2010.04.001

86. Kawabata Y, Nakajima N, Miyake H, *et al*. Venous Sinus Stenting for Transverse Sinus Stenosis Associated with Leptomeningeal Carcinomatosis in a Patient with Epidermal Growth Factor Receptor-Mutated Lung Cancer: A Case Report. *Am J Case Rep*. 2020;21:e918488. doi:10.12659/AJCR.918488

87. Keshary SR, Everett T, Alvarado AM, *et al*. Stent-assisted coiling of dural sinus diverticula: a case series. *J Neurointerv Surg*. 2021;neurintsurg-2020-016937. doi:10.1136/neurintsurg-2020-016937

88. Keshary SR, Everett T, Alvarado AM, et al. Stent-assisted coiling of dural sinus diverticula: a case series. In: Proceedings from the 9th Annual Meeting and 4th Annual Stroke Center Workshop (6M Conference); November 16-19, 2015; New York, NY, USA. doi:10.1159/000453053

89. Craven C, Patel NA, Khan AA, *et al*. Persistent CSF leak post spinal surgery and cerebrospinal fluid dynamic disturbances: cause or consequence? *Fluids Barriers CNS*. 2015;12(Suppl 1):P11. doi:10.1186/2045-8118-12-s1-p11

90. Khan A, Craven C, Toma A, *et al*. Persistent cerebrospinal fluid (CSF) leak post spinal surgery and cerebrospinal fluid dynamic disturbances: cause or consequence? *The Spine Journal*. 2016;16(4):S72. doi:10.1016/j.spinee.2016.01.081

91. Khan SU, Siddiqui MMR, Rahman KM, *et al*. Dural Arteriovenous Fistula - A Review. *Journal of Medicine*. 1970;11(2):159-166. doi:10.3329/jom.v11i2.5464

92. Kikuchi Y. A new concept and proposed treatment of the brain arteriovenous malformation (AVM). In: Proceedings from the 40th European Society of Neuroradiology Diagnostic and Interventional Annual Meeting; September 13-17, 2017; Malmö, Sweden. doi:10.1007/s00234-017-1872-5. Abstract 2.P4.
93. Kirsch M, Liebig T, Kühne D, *et al*. Endovascular management of dural arteriovenous fistulas of the transverse and sigmoid sinus in 150 patients. *Neuroradiology*. 2009;51(7):477-483. doi:10.1007/s00234-009-0524-9
94. Kumpe D, Seinfeld J. Dural sinus stenting for advanced pseudotumor cerebri with papilledema: Patient selection and results. In: Proceedings from the 7th Annual Meeting of the Society of NeuroInterventional Surgery; July 26-30, 2010. Carlsbad, California, USA. doi:10.1136/jnis.2010.003251.66. Abstract E-066.
95. Labeyrie MA, Fantoni M, Vever U, *et al*. Intracranial venous sinus stenting for the treatment of lateral sinus stenoses: An analysis of 200 patients. *Diagn Interv Imaging*. 2021;102(10):619-627. doi:10.1016/j.diii.2021.05.008
96. Labeyrie MA, Bedarida V, Vever U, *et al*. Venous sinus stenting after repair of skull base spontaneous cerebrospinal fluid leaks: A single-center retrospective cohort series with case-control analysis. *J Neuroradiol*. 2022;49(2):164-168. doi:10.1016/j.neurad.2021.07.002
97. Lenck S, Labeyrie MA, Vallee F, et al. Stent Placement for Disabling Pulsatile Tinnitus Caused by a Lateral Sinus Stenosis: A Retrospective Study. *Oper Neurosurg (Hagerstown)*. 2017;13(5):560-565. doi:10.1093/ons/opx026
98. Lenck S, Vallée F, Civelli V, *et al*. Assessment of blood flow velocities and venous pressures using a dual-sensor guidewire in symptomatic dural sinus stenoses. *J Neurosurg*. 2018;1-5. doi:10.3171/2017.12.JNS172364
99. Lenck S, Nouet A, Shotar E, *et al*. Transverse sinus stenting without surgical repair in idiopathic CSF rhinorrhea associated with transverse sinus stenoses: a pilot study. *J Neurosurg*. 2021;1-7. doi:10.3171/2021.5.JNS21894
100. Haider AS, Sumdani H, McCaslin J, *et al*. Aggressive Endovascular Management of Massive Dural Venous Sinus Thrombosis in the Setting of Acute Myeloid Leukemia. *Cureus*. 2019;11(1):e3891. doi:10.7759/cureus.3891
101. Levrier O, Métellus P, Fuentes S, *et al*. Use of a self-expanding stent with balloon angioplasty in the treatment of dural arteriovenous fistulas involving the transverse and/or sigmoid sinus: functional and neuroimaging-based outcome in 10 patients. *J Neurosurg*. 2006;104(2):254-263. doi:10.3171/jns.2006.104.2.254
102. Li K, Ren M, Meng R, *et al*. Dural Arteriovenous Fistula Formation Complicated Cerebral Venous Sinus Stenosis After Venous Sinus Stenting. *World Neurosurg*. 2018;120:400-402. doi:10.1016/j.wneu.2018.08.230

103. Li K, Ren M, Meng R, *et al*. Efficacy of stenting in patients with cerebral venous sinus thrombosis-related cerebral venous sinus stenosis. *J Neurointerv Surg*. 2019;11(3):307-312. doi:10.1136/neurintsurg-2018-014328
104. Liebig T, Henkes H, Brew S, *et al*. Reconstructive treatment of dural arteriovenous fistulas of the transverse and sigmoid sinus: transvenous angioplasty and stent deployment. *Neuroradiology*. 2005;47(7):543-551. doi:10.1007/s00234-005-1377-5
105. Lin YH, Lee CW, Liu HM. Clinical outcomes of adjunct sinus stenting in dural arteriovenous fistulas : Role of flow restoration in steno-occlusion and cortical venous reflux. *Clin Neuroradiol*. 2022;32(2):455-464. doi:10.1007/s00062-021-01031-3
106. Lopez-Rivera V, Inam M, Lekka E, *et al*. O-032 How low is enough? – Defining the threshold with venous gradient pressure for venous sinus stenting in intracranial hypertension patients with venous sinus stenosis. *Journal of NeuroInterventional Surgery*. 2020;12(Suppl 1):A22-A22. doi:10.1136/neurintsurg-2020-SNIS.32
107. Lu G, Shin JH, Song Y, *et al*. Stenting of symptomatic lateral sinus thrombosis refractory to mechanical thrombectomy. *Interv Neuroradiol*. 2019;25(6):714-720. doi:10.1177/1591019919852168
108. MacIntosh PW, Jain S, Moss HE, *et al*. A school of red herring. *Surv Ophthalmol*. 2014;59(6):664-670. doi:10.1016/j.survophthal.2014.01.010
109. Mapakshi S, Puli S, Zahid K, *et al*. A Case of Recurrent Cerebral Venous Sinus Thrombosis in a Patient with Ulcerative Colitis and Methyl Tetrahydrofolate Reductase Gene Mutation: 1764. *Am J Gastroenterol*. 2016;111:S845-846. doi:10.1136/neurintsurg-2020-SNIS.32
110. Mariniello G, Giamundo A, Donzelli R, *et al*. Intracranial hypertension due to meningioma of the unique transverse sinus. *Neuroradiol J*. 2013;26(2):209-212. doi:10.1177/197140091302600211
111. Martinez ML, Mekabaty AE, Luciano M, *et al*. Defining treatment options for patients with complex CSF disorders manifesting as mixed features of intracranial hypotension and hypertension. In: Proceedings from the Ninth Annual Meeting of the International Society for Hydrocephalus and CSF disorders; September 23–25, 2017; Kobe, Japan. doi:10.1186/s12987-017-0084-z. Abstract 112.
112. Matsumoto N, Kuramoto Y, Shinoda N, *et al*. A case of stenting for acute cerebral venous sinus thrombosis in the superior sagittal sinus. *Interv Neuroradiol*. 2016;22(6):709-710. doi:10.1177/1591019916663093
113. Michel P, Angelillo-Scherrer A, Maeder P, *et al*. Stenting as a treatment for exercise-induced intracranial hypertension from bilateral jugular vein obstruction. *Neurol Clin Pract*. 2016;6(2):e10-e13. doi:10.1212/CPJ.0000000000000200
114. Mokin M, Kan P, Abla AA, *et al*. Intravascular ultrasound in the evaluation and management of cerebral venous disease. *World Neurosurg*. 2013;80(5):. doi:10.1016/j.wneu.2012.04.004

115. Mollan SP, Davies B, Silver NC, *et al*. Idiopathic intracranial hypertension: consensus guidelines on management. *J Neurol Neurosurg Psychiatry*. 2018;89(10):1088-1100. doi:10.1136/jnnp-2017-317440
116. Morris A, Higgins JN, Axon PR. Fulminant intracranial hypertension as a result of otological surgery: case report and discussion of management. *J Laryngol Otol*. 2021;135(6):551-554. doi:10.1017/S0022215121001262
117. Murakami T, Toyota S, Suematsu T, *et al*. Balloon anchoring to traverse the sigmoid sinus for delivery of large-caliber venous stents: A technical note. *Interv Neuroradiol*. 2021;27(5):712-715. doi:10.1177/1591019921996885
118. Ohara N, Toyota S, Kobayashi M, *et al*. Superior sagittal sinus dural arteriovenous fistulas treated by stent placement for an occluded sinus and transarterial embolization. A case report. *Interv Neuroradiol*. 2012;18(3):333-340. doi:10.1177/159101991201800314
119. Owler BK, Parker G, Halmagyi GM, *et al*. Pseudotumor cerebri syndrome: venous sinus obstruction and its treatment with stent placement. *J Neurosurg*. 2003;98(5):1045-1055. doi:10.3171/jns.2003.98.5.1045
120. Owler BK, Allan R, Parker G, *et al*. Pseudotumour cerebri, CSF rhinorrhoea and the role of venous sinus stenting in treatment. *Br J Neurosurg*. 2003;17(1):79-83. doi:10.3109/02688690309177979
121. Owler BK, Parker G, Halmagyi GM, *et al*. Cranial venous outflow obstruction and pseudotumor Cerebri syndrome. *Adv Tech Stand Neurosurg*. 2005;30:107-174. doi:10.1007/3-211-27208-9_4
122. Oxley, T. STENTRODE First in Human Early Feasibility Study (SWITCH). ClinicalTrials.gov identifier: NCT03834857. Updated September 1, 2021. Accessed August 1, 2022. https://clinicaltrials.gov/ct2/show/NCT03834857
123. Oxley TJ, Yoo PE, Rind GS, *et al*. Motor neuroprosthesis implanted with neurointerventional surgery improves capacity for activities of daily living tasks in severe paralysis: first in-human experience. *J Neurointerv Surg*. 2021;13(2):102-108. doi:10.1136/neurintsurg-2020-016862
124. Patsalides A, Santillan A, Sundararajan SH, *et al*. Venous sinus stenting for the treatment of isolated pulsatile tinnitus: Results of a prospective trial. *Interv Neuroradiol*. 2021;27(2):266-274. doi:10.1177/1591019920974185
125. Pereira VM, Cancelliere NM, Najafi M, *et al*. Torrents of torment: turbulence as a mechanism of pulsatile tinnitus secondary to venous stenosis revealed by high-fidelity computational fluid dynamics. *J Neurointerv Surg*. 2021;13(8):732-737. doi:10.1136/neurintsurg-2020-016636
126. Qiu XY, Zhao PF, Ding HY, *et al*. Bone remodeling in sigmoid sinus diverticulum after stenting for transverse sinus stenosis in pulsatile tinnitus: A case report. *World J Clin Cases*. 2021;9(10):2320-2325. doi:10.12998/wjcc.v9.i10.2320
127. Quintas-Neves M, Freitas E, Amorim JM, *et al*. Venous Sinus Stenosis Causing Isolated Pulsatile Tinnitus. *Can J Neurol Sci*. 2019;46(5):591-592. doi:10.1017/cjn.2019.73

128. Radvany MG, Gomez J, Gailloud P. Intravascular ultrasound of the transverse sinus in two patients with pseudotumor cerebri: technical note. *J Neurointerv Surg*. 2011;3(4):379-382. doi:10.1136/jnis.2011.004663
129. Rangel-Castilla L, Siddiqui AH. Nuances of Endovascular Treatment of Transverse/Sigmoid Sinus Stenosis With Stenting Venoplasty in a Patient With Pseudotumor Cerebri: 2-Dimensional Operative Video. *Oper Neurosurg (Hagerstown)*. 2019;16(3):393-394. doi:10.1093/ons/opy174
130. Raper DMS, Buell TJ, Ding D, *et al*. A pilot study and novel angiographic classification for superior sagittal sinus stenting in patients with non-thrombotic intracranial venous occlusive disease. *J Neurointerv Surg*. 2018;10(1):74-77. doi:10.1136/neurintsurg-2016-012906
131. Rekik M, Ayadi O, Moalla KS, *et al*. Atypical visual impairment associated to cerebral sinus stenosis related to COVID-19 thrombosis. *J Neurol Sci*. 2021;429:118481. doi:10.1016/j.jns.2021.118481
132. Renieri L, Michelozzi C, Brinjikji W, *et al*. PTA Stent of Dural Sinuses in Brain DAVF : A Report of 4 Cases. *Clin Neuroradiol*. 2019;29(2):331-339. doi:10.1007/s00062-017-0652-2
133. Roggerone S, Traverse-Glehen A, Derex L, *et al*. Recurrent cerebral venous thrombosis revealing paraneoplastic angiitis in Hodgkin's lymphoma. *J Neurooncol*. 2008;89(2):195-198. doi:10.1007/s11060-008-9604-7
134. Rosenberg KI, Banik R. Pseudotumor cerebri syndrome associated with giant arachnoid granulation. *J Neuroophthalmol*. 2013;33(4):417-419. doi:10.1097/WNO.0b013e3182a5943b
135. San Millán D, Hallak B, Wanke I, *et al*. Dural venous sinus stenting as a stand-alone treatment for spontaneous skull base CSF leak secondary to venous pseudotumor cerebri syndrome. *Neuroradiology*. 2019;61(9):1103-1106. doi:10.1007/s00234-019-02251-8
136. Santos-Franco JA, Lee A, Nava-Salgado G, *et al*. Hybrid carotid stent for the management of a venous aneurysm of the sigmoid sinus treated by sole stenting. *Vasc Endovascular Surg*. 2012;46(4):342-346. doi:10.1177/1538574412442593
137. Shanmugarajah PD, Hodgson TJ, Higgins JNP, *et al*. POC23 Venous sinus stenting in a patient with multiple meningiomata—a case report. *Journal of Neurology, Neurosurgery & Psychiatry*. 2010;81(11):e40-e40. doi:10.1136/jnnp.2010.226340.93
138. Shastri RK, Chaudhary N, Pandey AS, *et al*. Venous Diverticula Causing Pulsatile Tinnitus Treated With Coil Embolization and Stent Placement With Resolution of Symptoms: Report of Two Cases and Review of the Literature. *Otol Neurotol*. 2017;38(9):e302-e307. doi:10.1097/MAO.0000000000001540
139. Signorelli F, Mahla K, Turjman F. Endovascular treatment of two concomitant causes of pulsatile tinnitus: sigmoid sinus stenosis and ipsilateral jugular bulb diverticulum. Case report and literature review. *Acta Neurochir (Wien)*. 2012;154(1):89-92. doi:10.1007/s00701-011-1202-3
140. Stark RM, Liu K, Crowley W, *et al*. Assessment of Intracranial Pressure During Venous Sinus Stenting. Journal of Neurological Surgery Part B: Skull Base. 2016;77(S 01). doi:10.1055/s-0036-1579868

141. Starke RM, Liu K, Durst C, *et al*. A prospective pilot study of intraparenchymal & intravenous cerebral pressure assessment during venous sinus stenting. In: Proceedings from the 2016 AANS Annual Scientific Meeting; April 30 – May 4, 2016 ; Chicago, IL, USA. doi:10.3171/2016.4.JNS.AANS2016abstracts. Abstract 668.
142. Su H, Li B, Wang J, *et al*. Headache attributed to cranial venous sinus stenting: A case series and literature review. *Cephalalgia*. 2019;39(10):1277-1283. doi:10.1177/0333102419847752
143. Sundararajan SH, Ramos AD, Kishore V, *et al*. Dural Venous Sinus Stenosis: Why Distinguishing Intrinsic-versus-Extrinsic Stenosis Matters. *AJNR Am J Neuroradiol*. 2021;42(2):288-296. doi:10.3174/ajnr.A6890
144. Szitkar B. A meningioma exclusively located inside the superior sagittal sinus responsible for intracranial hypertension. *AJNR Am J Neuroradiol*. 2010;31(6):E57-E58. doi:10.3174/ajnr.A2130
145. Tajiri H, Mori T, Iwata T, *et al*. Clinical and angiographic outcomes following balloon angioplasty and stenting of venous sinus occlusion coupled with dural arteriovenous fistulas. *Neuroradiology*. 2009;51(Suppl 1):S25-S97. doi:10.1007/s00234-009-0561-4
146. Takada S, Isaka F, Nakakuki T, *et al*. Torcular dural arteriovenous fistula treated via stent placement and angioplasty in the affected straight and transverse sinuses: case report. *J Neurosurg*. 2015;122(5):1208-1213. doi:10.3171/2014.12.JNS141374
147. Takahashi Y, Suda Y, Saito A, *et al*. Endovascular stenting of the superior sagittal sinus stenosis by meningioma invasion presenting intracranial hypertension; a case report. In: Proceedings from the 29th International Symposium on Cerebral Blood Flow, Metabolism and Function and the 14th International Conference on Quantification of Brain Function with PET; July 4-7, 2019 ; Yokohama Japan. doi:10.1177/0271678X19851020. Abstract PB03-G09.
148. Trivelato FP, Araújo JF, Dos Santos Silva R, *et al*. Endovascular treatment of pulsatile tinnitus associated with transverse sigmoid sinus aneurysms and jugular bulb anomalies. *Interv Neuroradiol*. 2015;21(4):548-551. doi:10.1177/1591019915590367
149. Troffkin NA, Graham CB 3rd, Berkmen T, *et al*. Combined transvenous and transarterial embolization of a tentorial-incisural dural arteriovenous malformation followed by primary stent placement in the associated stenotic straight sinus. Case report. *J Neurosurg*. 2003;99(3):579-583. doi:10.3171/jns.2003.99.3.0579
150. Tsumoto T, Miyamoto T, Shimizu M, *et al*. Restenosis of the sigmoid sinus after stenting for treatment of intracranial venous hypertension: case report. *Neuroradiology*. 2003;45(12):911-915. doi:10.1007/s00234-003-1112-z
151. Wang Z, Ding J, Chen J, *et al*. Cerebral venous sinus stenosis should not be neglected when cerebral artery stenosis is confirmed: a case report. *Int J Neurosci*. 2021;131(12):1237-1242. doi:10.1080/00207454.2020.1782901
152. Waser B, Wood HM, Mews P, *et al*. Transverse sinus stenting for treatment of papilloedema secondary to a large brain herniation into a dural venous sinus with associated tectal plate lesion:

Case report and literature review. *Interv Neuroradiol*. 2021;27(6):756-762. doi:10.1177/15910199211003451

153. Watanabe M, Semaan E, Majidi S, *et al*. Treatment of Cerebral Dural Sinus Stenosis Using Stent Placement: A Systematic Review (P07.273). Neurology. 2013;80(7 Supplement):P07.273-P07.273.
154. Weber W, Kis B, Esser J, *et al*. Endovascular Treatment of a Dural Arteriovenous Fistula of the Transverse Sinus by Recanalisation, Angioplasty and Stent Deployment. A Case Report and Follow-up. *Interv Neuroradiol*. 2003;9(1):65-69. doi:10.1177/159101990300900111
155. Winters HS, Parker G, Halmagyi GM, *et al*. Delayed relapse in pseudotumor cerebri due to new stenosis after transverse sinus stenting. *J Neurointerv Surg*. 2016;8(10):e41. doi:10.1136/neurintsurg-2015-011896.rep
156. Xu K, Yu T, Yuan Y, *et al*. Current Status of the Application of Intracranial Venous Sinus Stenting. *Int J Med Sci*. 2015;12(10):780-789. doi:10.7150/ijms.12604
157. Xu Y, Meng R, Rajah GB, *et al*. Long-term Outcomes of Cerebral Venous Sinus Stenosis Corrected by Stenting. *Curr Neurovasc Res*. 2019;16(1):77-81. doi:10.2174/1567202616666190206185133
158. Yang IH, Pereira VM, Lenck S, *et al*. Endovascular treatment of debilitating tinnitus secondary to cerebral venous sinus abnormalities: a literature review and technical illustration. *J Neurointerv Surg*. 2019;11(8):841-846. doi:10.1136/neurintsurg-2019-014725
159. Yang MS, Chen CC, Hung HC, *et al*. Angioplasty as the first-line therapeutic option for venous hypertension with outlet obstruction of dural sinus. *Neuroradiol J*. 2008;21(1):121-127. doi:10.1177/197140090802100118
160. Yeh PS, Wu TC, Tzeng WS, *et al*. Endovascular angioplasty and stent placement in venous hypertension related to dural arteriovenous fistulas and venous sinus thrombosis. *Clin Neurol Neurosurg*. 2010;112(2):167-171. doi:10.1016/j.clineuro.2009.10.018
161. Zhang K, Gao BL, Zhu LF, *et al*. Endovascular Recanalization of Occluded Dural Sinus in Patient with Dural Arteriovenous Fistulas: Case Report and Literature Review. *World Neurosurg*. 2018;114:269-273. doi:10.1016/j.wneu.2018.03.140
162. Zheng H, Zhou M, Zhao B, *et al*. Pseudotumor cerebri syndrome and giant arachnoid granulation: treatment with venous sinus stenting. *J Vasc Interv Radiol*. 2010;21(6):927-929. doi:10.1016/j.jvir.2010.02.018
163. Zhu X, Gong X, Zhang B, *et al*. Application of Transcranial Color-coded Duplex Sonography in the Diagnosis and Management of Straight Sinus Thrombosis With Dural Arteriovenous Fistulae: A Case Report. *Neurologist*. 2020;25(6):180-186. doi:10.1097/NRL.0000000000000293
164. Zilani G, Pereira EA, Baig F, *et al*. Venoplasty and stenting of a jugular foramen meningioma. *Br J Neurosurg*. 2009;23(5):557-560. doi:10.1080/02688690903254368
165. Zenteno M, Murillo-Bonilla L, Martínez S, *et al*. Endovascular treatment of a transverse-sigmoid sinus aneurysm presenting as pulsatile tinnitus. Case report. *J Neurosurg*. 2004;100(1):120-122. doi:10.3171/jns.2004.100.1.0120
